# An Examination of the Effects of Propolis and Quercetin in a Rat Model of Streptozotocin-Induced Diabetic Peripheral Neuropathy

**DOI:** 10.3390/cimb46030128

**Published:** 2024-03-02

**Authors:** Sibel Türedi, Hakim Çelik, Şeyda Nur Dağlı, Seyhan Taşkın, Uğur Şeker, Mustafa Deniz

**Affiliations:** 1 Department of Histology and Embryology, Faculty of Medicine, Harran University, Şanlıurfa 63050, Turkey; 2 Department of Physiology, Faculty of Medicine, Harran University, Şanlıurfa 63050, Turkey; hakimcell@gmail.com (H.Ç.); sehan_taskin@yahoo.com (S.T.); 3 Department of Physiology, Faculty of Medicine, İstinye University, İstanbul 34000, Turkey; seyda_dgl@hotmail.com; 4 Department of Histology and Embryology, Faculty of Medicine, Mardin Artuklu University, Mardin 47100, Turkey; seker.ugur.tr@gmail.com; 5 Department of Anatomy, Faculty of Medicine, Harran University, Şanlıurfa 63050, Turkey; denizmf@hotmail.com

**Keywords:** diabetic peripheral neuropathy, propolis, quercetin, regenerative medicine, TEM microscope, rat

## Abstract

The purpose of this study was to reveal the combined effects of propolis (P) and quercetin (Q) against diabetic peripheral neuropathy developing with streptozotocin-induced diabetes in rats. Sixty-four adult male rats were divided into eight equal groups: control, P (100 mg/kg/day), Q (100 mg/kg/day), P + Q (100 mg/day for both), diabetes mellitus (DM) (single-dose 60 mg/kg streptozotocin), DM + P, DM + Q, and DM + P + Q. The rats were sacrificed, and blood and sciatic nerve tissues were collected. Blood glucose and malondialdehyde (MDA) levels increased, while IL-6 and total antioxidant status decreased in the DM group (*p* = 0.016 and *p* = 0.047, respectively). Ultrastructural findings showed degeneration of the axon and myelin sheath. The apoptotic index (AI %), TNF-α, and IL-1β immunopositivity increased significantly in the DM group (*p* < 0.001). Morphological structures approaching those of the controls were observed in the DM + P, DM + Q, and DM + P + Q groups. Morphometric measurements increased markedly in all treatment groups (*p* < 0.001), while blood glucose and MDA levels, AI (%), TNF-α, and IL-1β immunopositivity decreased. In conclusion, the combined effects of propolis and quercetin in diabetic neuropathy may provide optimal morphological protection with neuroprotective effects by reducing hyperglycemia, and these may represent a key alternative supplement in regenerative medicine.

## 1. Introduction

Neuropathy, the most widespread complication of diabetes, indicates the presence of a condition requiring urgent attention [1]. In the absence of successful intervention, it is predicted that one-third of the 9.7 billion world population by 2050 will be diabetics, and that half of these will also have neuropathy [2].

Diabetic peripheral neuropathy (DPN) is a neurodegenerative disease that begins distally in the lower extremities, is characterized by pain and significant morbidity, that targets the sensory neurons, autonomic axons, and subsequently the motor axons, and in which loss of structural and sensory function of the peripheral nervous system is observed [3,4]. Although the pathogenesis of DPN appears to involve a complex pathway, the likely mechanisms include oxidative stress, mitochondrial dysfunction, advanced glycation end products, the polyol pathway, and protein kinase C pathways. Schwann cell dysfunction has also been shown to play an important role in the pathogenesis of DPN, such as apoptosis, lipid metabolism abnormality, oxidative stress, inflammatory reactions, and endoplasmic reticulum stress. Moreover, glycemic variability (glucotoxicity) has also been implicated in the pathogenesis of diabetic complications and has been described as a probable independent risk factor for DPN [3,5,6]. Current approaches concerning DPN therefore focus on glycemic control, lifestyle modifications, and drug-based management [7]. Additionally, the use of antioxidants derived from micronutrients as complementary drugs in the treatment of DM is widely regarded as capable of reducing chronic complications associated with reactive oxygen species (ROS) that may develop in the long term [8].

STZ-induced diabetes is a relevant model of endogenous chronic oxidative stress due to the resulting hyperglycemia. The development of hyperglycemia in rats following STZ injection is primarily due to direct pancreatic beta cell destruction and resulting insulin deficiency [9]. Propolis is a unique resin-containing drug collected by honeybees from the leaves and shoots of plants and trees, which has various medical benefits and has enjoyed worldwide popularity for centuries [10]. Although research on propolis and its recommended dose is still ongoing, it has been reported that the safe dose in animal experiments and generally observed healthy humans is 70 mg/day. However, some studies have found that it was safe to give 150 mg of propolis daily [11]. Numerous experimental studies have reported that propolis and its components exhibit beneficial biomedical properties, such as antidiabetic, anti-inflammatory, antioxidant, anticancer, immunomodulatory, antibacterial, and antifungal activities [8,10,12]. Quercetin (3,3′,4′,5,7-pentahidroksiflavon) is a flavonoid present in numerous fruits and vegetables, including onions, broccoli, green tea, apples, ginkgo biloba, and yellow centaury, [13] the daily intake of quercetin varies between 5 and 40 mg, and it has been shown that the daily level reaches 200–500 mg in those who consume plenty of fruits and vegetables [14]. Also, it has antioxidant, anti-inflammatory, antiapoptotic, neuroprotective, and hepatoprotective effects [13].

The scientific community is still largely skeptical concerning the complementary effects of propolis and quercetin, despite the positive results on blood glucose, inflammatory markers, apoptosis, and oxidative stress observed in studies [8,13]. In previous studies investigating the effects of propolis and quercetin on various tissues at different doses and for different periods of time, it was observed that a daily dose of 100 mg/kg had an ameliorative effect [15,16,17]. Therefore, to investigate the effects on the neuropathy complication of DM in the sciatic nerve, the daily dose range of 100 mg was chosen. The purpose of this study was to reveal the effects of the separate and combined oral administration of quercetin and propolis, frequently consumed and easily available in daily life, on DPN in streptozotocin (STZ)-induced diabetic rats using ultrastructural, immunohistochemical, and biochemical parameters.

## 2. Materials and Methods

### 2.1. Ethical Procedures and Animals

Sixty-four healthy male Wistar albino rats (age 12–16 weeks, weight 300–400 g) were obtained from the Harran University (HRU) Experimental Animals Application and Research Center (HRU-HDAM) (Şanlıurfa, Türkiye). This study commenced following the receipt of approval from the HRU animal experiments local ethical committee (HADYEK) (study protocol license no. 2020/006/17). All rats were housed under standard laboratory conditions at 22 ± 2 °C, in 50% ± 10 humidity in a 12-h light:12-h dark cycle throughout the experimental period. All rats were also given standard laboratory chow and ad libitum access to water during the experiment. All animals received human care according to the criteria outlined in the “Guide for the Care and Use of Laboratory Animals” published by the National Institutes of Health.

### 2.2. Experimental Design

Sixty-four male Wistar albino rats (age 12–16 weeks, weight 300–350 g) were randomly assigned into eight groups as described below:

Control group (n:6): These animals were sacrificed at the end of the experimental period following a single dose of 0.1 molar (pH: 4.5) citrate buffer via the intraperitoneal route (i.p.).

Propolis (P) group (n:6): The rats were administered 100 mg/kg propolis per day by oral gavage, for the desired specific volume of the substances, throughout the experimental period, for 28 days [10].

Quercetin (Q) group (n:6): The rats were administered 100 mg/kg quercetin per day by oral gavage, for the desired specific volume of the substances, throughout the experimental period, for 28 days [16].

Propolis + quercetin (P + Q) group (P + Q) (n:6): The rats in this group received 100 mg/kg propolis and 100 mg/kg quercetin, respectively, per day by oral gavage, for the desired specific volume of the substances, throughout the experimental period, for 28 days.

DM group (n:10): The rats in this group received a single dose of 60 mg/kg STZ via the intraperitoneal route (i.p.) dissolved in 1 milliliter of 0.1 molar (pH: 4.5) citrate buffer. Blood sugar was measured from tail vein blood using a glucometer (Call^®^ Plus Blood Glucose Monitoring System, Acon Laboratories Inc., San Diego, CA, USA) after 72 h, and rats with blood sugar levels exceeding 300 mg/dl were regarded as diabetic [9].

DM + propolis (DM + P) group (n:10): Rats with DM induced by 60 mg/kg STZ (a single dose, i.p.) received, after accepting the DM model, 100 mg/kg propolis per day by oral gavage, for the desired specific volume of the substances, throughout the experiment, for 28 days.

DM + quercetin (DM + Q) group (n:10): Rats with DM induced by 60 mg/kg STZ (a single dose, i.p.) received, after accepting the DM model, 100 mg/kg quercetin per day by oral gavage, for the desired specific volume of the substances, throughout the experiment, for 28 days.

DM + propolis + quercetin (DM + P + Q) group (n:10): Rats with DM induced by 60 mg/kg STZ (a single dose, i.p.) received, after accepting the DM model, 100 mg/kg propolis and 100 mg/kg quercetin per day, respectively, by oral gavage, for the desired specific volume of the substances, throughout the experiment, for 28 days.

Following the establishment of the STZ-based DM model, blood sugar levels were monitored weekly during the four-week experimental period. At the end of that period, all rats were placed in the prone position under deep anesthesia (ketamine 90 mg/kg plus xylazine 10 mg/kg) (Ketalar^®^, Eczacıbaşı Co., Istanbul, Turkey; Rompun 2%, Bayer Healthcare LLC, Istanbul, Turkey). The right lateral thigh was opened with a surgical incision and a clip was applied. A unilateral muscular incision extending from the greater trochanter to the mid-thigh was then made, and the right sciatic nerve was exposed, severed proximally and distally, and removed. Sciatic nerve tissues and blood specimens collected from the experimental groups were set aside for biochemical evaluation and light and electron microscopic examination.

### 2.3. Evaluation of Rat Sciatic Nerves: Histopathological Analysis and Transmission Electron Microscopy

Tissue samples were obtained 0.5 cm distally to the sciatic nerves. The sciatic nerve tissues from all the study groups were fixed in 10% neutral formaldehyde solution for histopathological examination using routine histological procedures and embedded in paraffin. Sections 5 µm in thickness were then taken from the paraffin blocks using a semi-automatic rotary microtome (Thermo Shandon Finesse ME+ Microtome, Runcorn, UK) and stained with Masson’s trichrome (Trichrome Masson Stain Kit—Sigma Aldrich, St. Louis, MO, USA, CAS Number: HT15-1KT). All findings and evaluations were recorded onto a computer using a Zeiss Axioscope II (Carl Zeiss Microscopy GmbH, Göttingen, Germany) microscope and photographed with a Zeiss Axiocam MRc camera attachment (Carl Zeiss MicroImaging GmbH, Göttingen, Germany). Osmium tetroxide (Sigma-Aldrich Chemie GmbH, Saint Louis, MO, USA, CAS Number: 20816-12-0) induces good fixation of myelinated axons at the peripheral nerve, and toluidine blue was used for staining, as previously described [18]. Briefly, tissue samples taken 0.5 cm distally to the sciatic nerve injury were fixed for 4 h with 2.5% glutaraldehyde (Merck, Schuchardt OHG, Saint Louis, MO, USA, CAS Number: 111-30-8) in a 0.4 M phosphate-buffered saline (PBS) solution (pH 7.4) at 4 °C. Post-fixation, tissue samples were fixed for 1 h in 1% OsO_4_ at 4 °C and subsequently passed through an increasing alcohol series (50%–70%–90%–96%); prior to embedding in and subsequent transfer to propylene oxide, samples were embedded in epoxy resin [18]. Semi-fine sections were cut into 500 nm sections using a Leica Reichert Ultracut R ultramicrotome (Leica Microsystems GmbH, Wetzlar, Germany) and were stained with 1% toluidine blue. Morphometric analysis of the sciatic nerve sections, the number of myelinated nerve fibers, the myelin sheath thickness, and the nerve fiber diameter were measured in five distinct areas from each section [15]. All evaluations were recorded onto a computer using a Zeiss Axioscope II (Carl Zeiss Microscopy GmbH, Göttingen, Germany) microscope and photographed with a Zeiss Axiocam MRc camera attachment (Carl Zeiss MicroImaging GmbH, Göttingen, Germany). For transmission electron microscopic examinations, 50 nm ultra-fine sections were placed onto 200–300 mesh copper grids using a double contrast method by 2.5% uranyl–acetic acid and lead citrate [19]. The sections were then examined under a transmission electron microscope (JEOL-JEM-1010, Tokyo, Japan).

### 2.4. TUNEL Assay

Apoptosis in sciatic nerve tissues was analyzed using the terminal deoxynucleotidyl transferase (TdT) deoxyuridine triphosphate nick end labeling assay (TUNEL) method. Sections 5 μm in thickness were taken from the paraffin blocks and subjected to standard deparaffinization. TUNEL staining was performed using an In Situ cell Death Detection Kit (Roche, Mannheim, Germany, CAS Number: 11684817910) in accordance with the manufacturer’s instructions. Apoptosis evaluation from the TUNEL-stained slides was performed using a light microscope (Carl Zeiss Microscopy GmbH, Göttingen, Germany) at a magnification of ×400. Homogeneously stained TUNEL (+) Schwann cells with no necrotic areas were defined as apoptotic. Normal and apoptotic cells were recorded by counting 100 Schwann cells in five areas in each tissue at a magnification of ×400. The percentage of apoptotic nuclei [apoptotic index (AI)] was calculated as apoptotic nuclei/total nuclei counted × 100% [18].

### 2.5. Immunohistochemical Staining

Sections 5 µm in thickness were taken from the paraffin-embedded blocks and deparaffinized. After washing, they were next washed in PBS buffer solution for 5 min. The sections were subsequently boiled in citrate buffer (pH: 6.0) and subjected to antibody retrieval. Specimens washed in PBS were next subjected to peroxidase blocking in 3% H_2_O_2_ solution. TNF-α (Santa Cruz Biotechnology Inc., Heidelberg, Germany, CAS Number:sc-52746) and IL-1β (Santa Cruz Biotechnology Inc., Heidelberg, Germany, CAS Number:sc-52012) antibodies diluted to 1:100 were next dropped onto the specimens and left to incubate at +4 °C. The subsequent procedures were performed using secondary antibody kits (Thermo Scientific, Waltham, MA, USA, CAS number: TP-060-HL), and all steps were carried out in line with the manufacturer’s instructions. A 3,3′-diaminobenzidine (DAB) chromogen kit was employed (Sigma-Aldrich, St. Louis, MO, USA, CAS Number D3939). The specimens were counterstained with Mayer’s hematoxylin, covered with Entellan, and examined under a light microscope. All evaluations were recorded onto a computer using a Zeiss Axioscope II (Carl Zeiss Microscopy GmbH, Göttingen, Germany) microscope or image analysis software and photographed with a Zeiss Axiocam MRc camera attachment (Carl Zeiss MicroImaging GmbH, Göttingen, Germany). For immunohistochemical analysis of sciatic nerve tissue, five different areas were randomly selected in each section. TNF-α and IL-1β positivity were defined as represented by a brown color, and numerical evaluation was applied. Immunohistochemical labeling was scored based on both the intensity and distribution of specific staining and semiquantitative estimates. The intensity of staining was reported based on the H-score method, using 0 for negative staining, 1+ for weak staining, 2+ for moderate staining, and 3+ for strong staining. The formula given below produces an H-score in the range of 0–300, where 300 equals 100% of strongly stained positive cells: H-score = (% of cells stained at intensity category 1 × 1) + (% of cells stained at intensity category 2 × 2) + (% of cells stained at intensity category 3 × 3 [20].

### 2.6. Biochemical Analysis

Biochemical analyses in serum samples: Following the sacrifice of the experimental animals under deep anesthesia, blood samples were drawn from the heart using a syringe in a gel tube and then centrifuged at 1500× *g* for 10 min at 4 °C (Hettich, Westphalia, Germany) to separate the serum samples. The separated sera were stored in Eppendorf tubes at −86 °C in a deep freezer until the time of the study when they were thawed to room temperature. The levels of TNF-α and IL-6 in the serum samples were determined using commercially available ELISA kits (Elabscience Biotechnology Co., Ltd., Wuhan, China, CAS numbers E-EL-R2856 and E-EL-R0015, respectively), while total antioxidant status (TAS) and total oxidant status (TOS) levels were assessed using commercially available colorimetric measurement kits developed by Erel [21,22] (Rel Assay Diagnostics, Gaziantep, Türkiye, CAS numbers AK20114A and AK201260, respectively), all according to the manufacturer’s instructions. Absorbances were read using a Varioskan Lux microplate reader system (Thermo Scientific, Waltham, MA, USA), and the results were calculated based on standard concentrations. The oxidative stress index (OSI) was calculated as the percentage of (TOS) to TAS values. For this calculation, the unit of TAS (mmol Trolox Eqv./L) was converted to μmol Trolox Eqv./L. The OSI (AU) = (TOS, μmol H_2_O_2_ Eqv./L)/(TAS, μmol Trolox Eqv./L) × 100 formula was used, and the results were calculated in arbitrary units (AUs) [23].

Biochemical analyses in sciatic tissue: After the experimental animals were sacrificed under deep anesthesia, the collected sciatic nerve tissues were stored in empty Eppendorf tubes at −86 °C in a deep freezer until being used for biochemical analyses. On the day of the study, the sciatic tissue samples were removed from the freezer and dissected into smaller pieces using a scalpel. They were then weighed and transferred to 1.5 mL empty Eppendorf tubes. PBS (pH: 7.4) was added in a volume 10 times that of the tissue weight in a cold environment. The sciatic nerve tissues were homogenized according to the study protocol using a ball-bearing homogenization device (Retsch MM 400, Haan, Germany) at 28 Hz for 2.5 min. The resulting homogenates were centrifuged at 1500× *g* for 10 min at 4 °C to yield the supernatants. The protein quantities in the supernatants were measured using the BCA method. SOD, CAT, and GSH levels were determined using commercially available ELISA kits (Elabscience Biotechnology Co., Ltd., Wuhan, China, CAS numbers E-EL-R1425, E-BC-K031-M, and E-EL-0026, respectively). TAS and TOS levels were assessed using commercially available colorimetric measurement kits developed by Erel [20,21] (Rel Assay Diagnostics, Gaziantep, Türkiye, CAS numbers AK20114A and AK201260, respectively), all in accordance with the manufacturers’ instructions. Absorbances were read using the Varioskan Lux microplate reader system (Thermo Scientific, Waltham, MA, USA), and the results were calculated based on both standard and protein concentrations. The oxidative stress index (OSI) was calculated as the ratio of TOS to TAS values. For this calculation, the unit of TAS (mmol Trolox Eqv./mg protein) was converted to μmol Trolox Eqv./mg protein. The results were determined in AUs using the formula OSI (AU) = (TOS, μmol H_2_O_2_ Eqv./mg protein)/(TAS, μmol Trolox Eqv./mg protein) × 100 [23].

MDA measurement in the supernatant and serum specimens: MDA levels in the supernatant and serum specimens were determined using a standard thiobarbituric acid reactive substances (TBARS) measurement method [24]. MDA measurement using thiobarbituric acid (TBA) permits the determination of lipid peroxidation, the major biomarker of oxidative stress. The reaction mixture consisted of 100 µL of a specimen, 200 µL 20% TBA, and 600 µL 1% orthophosphoric acid. The measurement relies on the formation of a “pink”-colored pigment that can be extracted in butanol in an acidic and hot environment (100 °C) and that is absorbable at 530 nm. The TBARS concentration was calculated using the molar excitation coefficient (1.56 × 10^5^ M^−1^ cm^−1^). Serum data µmol/mL and supernatant data were expressed as nmol/mg protein.

### 2.7. Statistical Analysis

All statistical analyses were performed using SPSS version 25.0 software (Statistical Package for the Social Sciences, IBM SPSS Inc., Chicago, IL, USA). One-way ANOVA analysis of variance was used for one-way multiple comparisons of blood glucose levels, body weight morphometric analyses, the apoptotic index, and immunohistochemical damage scoring between the groups. Post hoc dual comparisons between groups exhibiting significant values were evaluated with a Tukey HSD test. For the biochemical analyses, intergroup one-way multiple comparisons were carried out using Kruskal–Wallis H analysis of variance, and the Bonferroni test and correction were used for post hoc within-group paired comparisons. Statistical significance for all tests was set at *p* < 0.05. Mean ($\bar{x}$) plus standard deviation (±) (SD) values were used.

## 3. Results

### 3.1. Changes in Body Weight and Blood Sugar Levels

Changes in body weight for all groups throughout the four-week experimental period are shown in Table 1. Body weight in the DM group decreased significantly compared with the control group (*p* < 0.001). Significant decreases were also observed in the DM + Q and DM + P + Q groups compared with the DM group (*p* = 0.001 and *p* < 0.001, respectively). One particularly noteworthy finding was that body weight in the combined treatment group (DM + P + Q) was close to that in the control group, with no statistically significant difference between them. In terms of clinical signs, the gastrointestinal tract was adversely affected in the groups receiving propolis, and some degree of diarrhea was observed. The groups’ weekly mean blood glucose levels over the four-week study period are shown in Table 1. Baseline blood glucose levels in the DM group in our STZ-based model were significantly higher on all four weeks compared with the control group (*p* < 0.05). Blood glucose levels in the DM + P, DM + Q, and DM + P + Q groups decreased significantly in the third and fourth weeks compared with the DM group (*p* < 0.05).

### 3.2. Histopathological and Morphometric Observations

**Figure 1:**
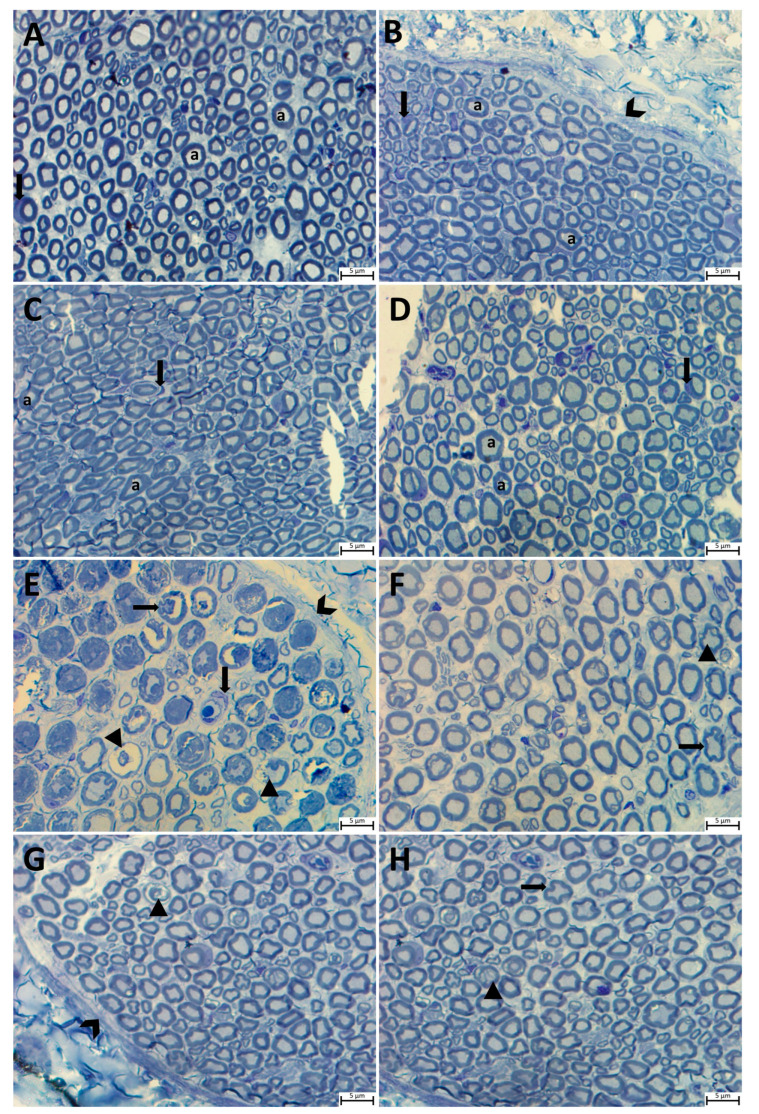
Light micrograph of transverse semi-thin sections of rat sciatic nerves. Notable features are indicated on the figure as follows: perineurium (chevrons), axon (for all axon; a), Schwann cells (for all Schwann cells; down arrow), myelin sheath and axon degeneration (arrowhead), and myelin sheath invaginated into the axon and disorganization (left arrow). (**A**); Control group, (**B**); propolis (P) group (**C**); quercetin (Q) group, (**D**); P + Q group, (**E**); diabetes mellitus (DM) group, (**F**); DM + P group, (**G**); DM + Q group, and (**H**); DM + P + Q group (toluidine blue magnification, ×100).

**Figure 2:**
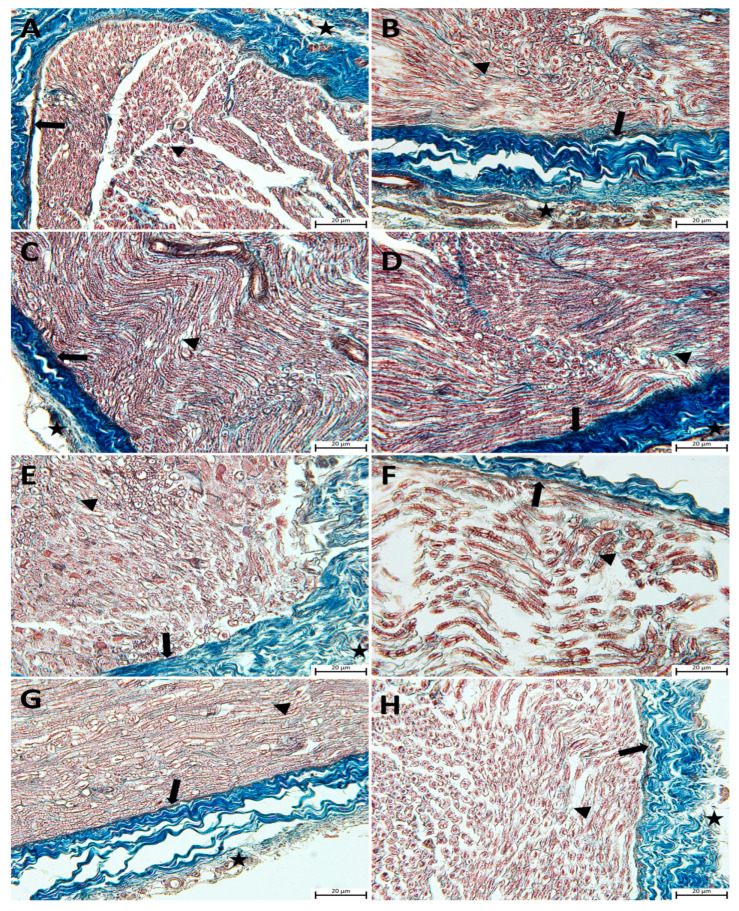
Light micrographs following Masson’s trichrome staining on sciatic nerve tissue sections and representative images of the results from each group are presented. Epineurium (star), perineurium (arrow), endoneurium (arrowhead). (**A**); Control group, (**B**); propolis (P) group (**C**); quercetin (Q) group, (**D**); P + Q group, (**E**); diabetes mellitus (DM) group, (**F**); DM + P group, (**G**); DM + Q group, and (**H**); DM + P+ Q group (magnification, ×40).

Histopathological examinations of sciatic nerve tissues from all the study groups were carried out using Masson’s trichrome and with toluidine blue staining in semi-thin sections. The light microscopic evaluation revealed a normal morphology in semi-thin sciatic nerve sections from the control, P, Q, and P + Q groups. A perineurium connective tissue layer was observed around the axon bundles in a unifascicular structure, surrounded by the epineurium and collected together in the form of fascicles. The density of myelinated axons in the nerve fascicle was particularly remarkable (Figure 1A–D and Figure 2A–D). In the DM group, the sciatic nerve was surrounded externally by an epineurium consisting of fibrous connective tissue, with a slight loss of integrity in the perineurium (Figure 2E). In semi-thin sections, the axon and myelin sheath in myelinated axons were entirely degenerated, and the concentric lamellar structure of the myelin sheath was severely impaired, with separations also being observed. Pronounced axonal swelling, vacuoles of differing sizes between the myelin sheath, and occasional invagination of the myelin sheath into the axon were observed, with some axons being entirely degenerated (Figure 1E). Examination of sciatic nerve tissues from the DM + P, DM + Q, and DM + P + Q groups showed that the integrity of connective tissue was completely preserved (Figure 2F–H), regeneration in nerve fibers occurred at the end of the 28-day experimental period, and axonal and myelin sheath structures were included in this process. A small amount of degeneration in the axon and myelin sheath was present in the DM + P group in particular. When the treatment groups were compared among themselves, the DM damage findings in the combined propolis and quercetin administration group (DM + P + Q) improved markedly, regeneration was active in both myelinated and unmyelinated nerve fibers, and morphological characteristics were close to those of the control group (Figure 1D–F).

**Figure 3:**
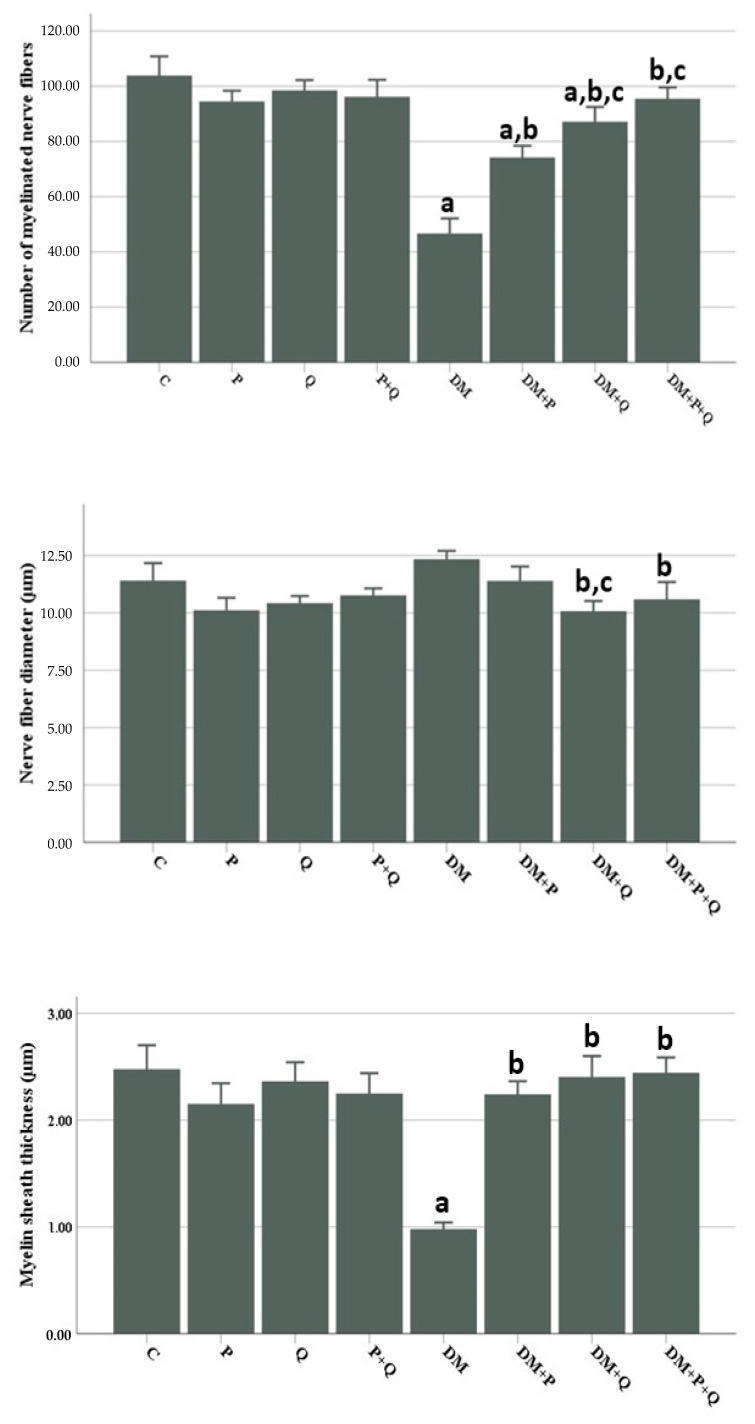
Histomorphometric parameters of rat sciatic nerves. Data are presented as mean ± SD ^a^; *p* < 0.05 compared with the control group, ^b^; *p* < 0.05 compared with the DM group, ^c^; *p* < 0.05 compared with the DM + P group. C; control, P; propolis, Q; quercetin, DM; diabetes mellitus.

Morphometric examination of semi-thin sections revealed a significant decrease in myelinated nerve fiber numbers and myelin sheath thickness in the DM group compared with the control group (*p* < 0.001), while the nerve fiber diameter increased to close to that of the control group. Nerve fiber diameters decreased markedly in the DM + Q and DM + P + Q groups compared with the DM group (*p* < 0.001 for both), while the myelin sheath thickness and numbers of myelinated nerve fibers increased significantly in the DM + P, DM + Q, and DM + P + Q groups (*p* < 0.001). When the treatment groups were compared among themselves, the most marked improvement in morphometric analysis was observed in the combined treatment group (DM + P + Q) (Figure 3).

### 3.3. Transmission Electron Microscopy Observation Results for Sciatic Nerve Tissue

**Figure 4:**
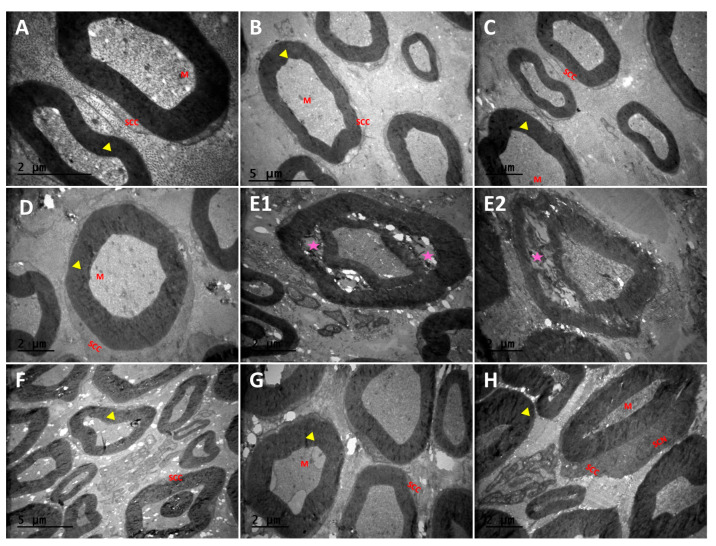
Transmission electron micrographs (TEMs) of rat sciatic nerves. Myelinated nerve fibers (yellow arrowhead) and separation in the concentric lamellae of the myelin sheath and damaged axons (purple color star), SCC; Schwann cells, SCN; Schwann cell nucleus, M; mitochondria. (**A**); Control group, (**B**); propolis (P) group (**C**); quercetin (Q) group, (**D**); P + Q group, (**E1**,**E2**); diabetes mellitus (DM) group, (**F**); DM + P group, (**G**); DM + Q group, and (**H**); DM + P+ Q group. (Magnification, (**A**); ×20,000, (**B**,**F**); ×6000, (**C**–**E1**,**G**,**H**); ×10,000, and (**E2**); ×12,000).

Transmission electron microscopy confirmed the structural and morphological findings observed under optic microscopy. Accordingly, axonal and myelination structures in the sciatic nerve were normal in the control P, Q, and P + Q groups, with numerous myelinated axons being observed (Figure 4A–D). Atrophic axons with spaces between them, areas of demyelination, and separation of openings in the myelin sheath were observed in the DM group (Figure 4(E1,E2)). However, in the DM + P, DM + Q, and DM + P + Q groups, the areas of demyelination observed in the DM group were converted to remyelination, the structure of the myelin sheath was preserved, and the axonal morphology and endoneurial area structure were close to those of the control group (Figure 4F–H).

### 3.4. Evaluation of Immunohistochemistry and TUNEL Staining Results

**Figure 5:**
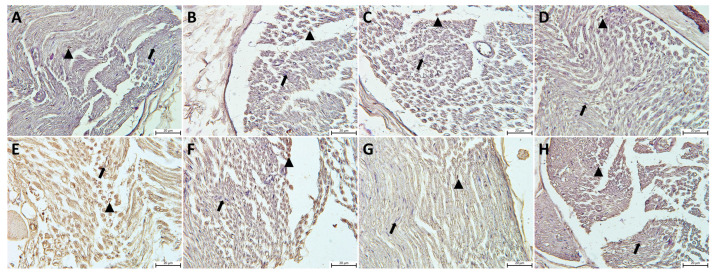
Nuclear changes suggestive of apoptosis were observed in TUNEL staining sections under light microscopy at a magnification of ×400. Apoptotic Schwann cell nucleus (arrowhead), normal Schwann cell nucleus (arrow). (**A**); Control group, (**B**); propolis (P) group (**C**); quercetin (Q) group, (**D**); P + Q group, (**E**); diabetes mellitus (DM) group, (**F**); DM + P group, (**G**); DM + Q group, and (**H**); DM + P + Q group.

**Figure 6:**
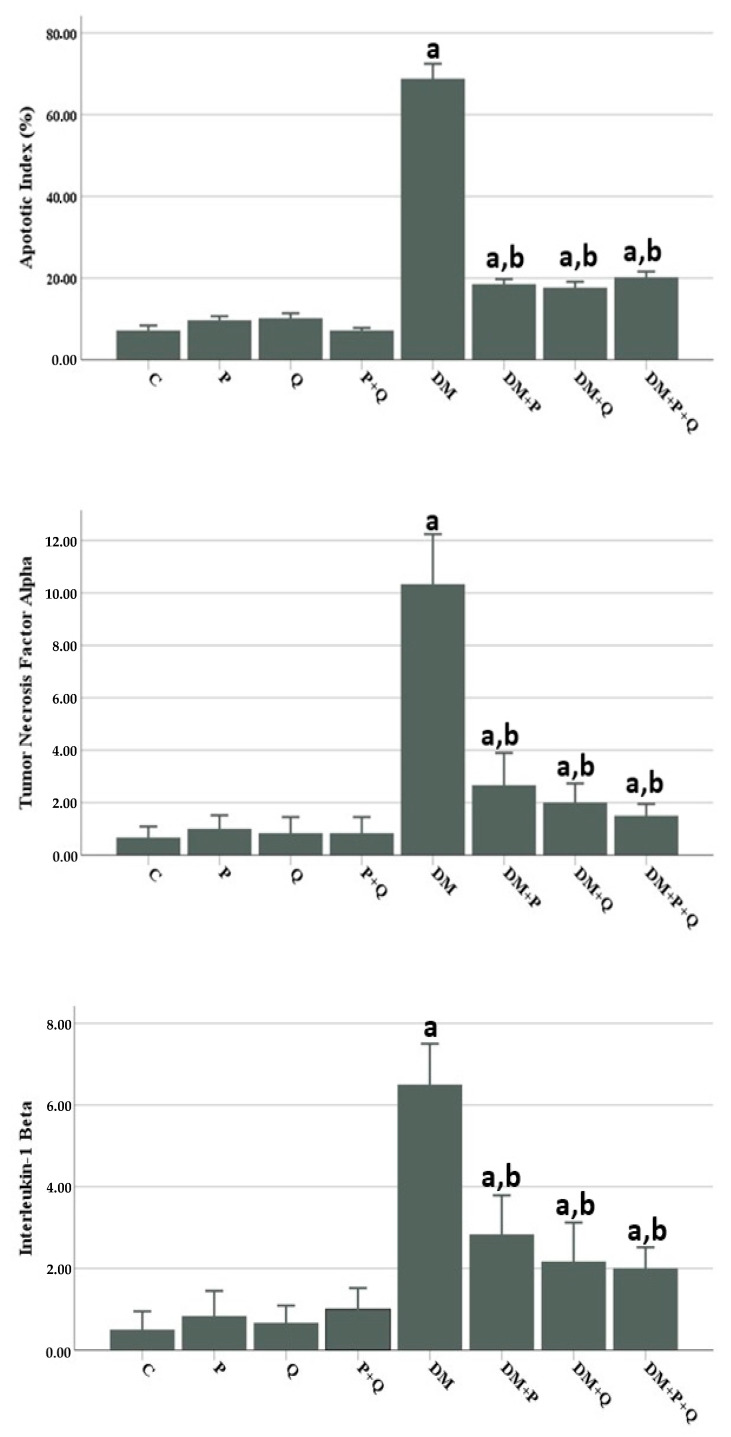
Apoptotic index (%) and immunohistochemical staining for tumor necrosis factor (TNF-α) and interleukin-1β (IL-1β) expression in the sciatic nerve samples. Data are presented as mean ± SD ^a^; *p* < 0.05 compared with the control group, ^b^; *p* < 0.05 compared with the DM group. C; control, P; propolis, Q; quercetin, DM; diabetes mellitus.

**Figure 7:**
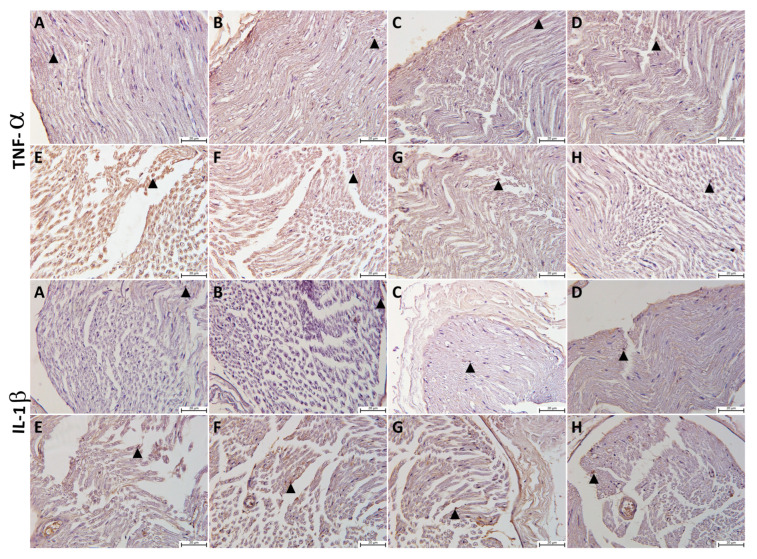
The immunohistochemical staining for tumor necrosis factor (TNF-α) and interleukin-1β (IL-1β) expression in the sciatic nerve samples of the different study groups: (**A**); control group, (**B**); propolis (P) group (**C**); quercetin (Q) group, (**D**); P + Q group, (**E**); diabetes mellitus (DM) group, (**F**); DM + P group, (**G**); DM + Q group, and (**H**); DM + P+ Q group. TNF-α- and IL-1β-positive staining (brown) (arrowhead) (magnification, 40×).

The presence of apoptosis was examined in TUNEL-stained sections under light microscopy for Schwann cells at a magnification of ×400. Apoptosis, Apoptotic index (AI) values and TNF-α and IL-1β immunopositivity H-scores in sciatic nerve tissues from all the study groups are shown in Figure 5 and Figure 6. Based on the study findings, no significant increase was determined in AI values or TNF-α and IL-1β immunopositivity in sciatic tissue from the P, Q, and P + Q groups compared with the control group (*p* > 0.05 for all) (Figure 5A–D and Figure 6. However, AI values and TNF-α and IL-1β immunopositivity increased significantly in the DM group compared with the control group (*p* < 0.001) (Figure 5E and Figure 6). AI values and TNF-α and IL-1β immunopositivity decreased markedly in the DM + P, DM + Q, and DM + P + Q groups compared with the DM group (*p* < 0.001 for all) (Figure 5F–H and Figure 7F–H).

### 3.5. Biochemical Parameter Findings

According to the biochemical analyses performed using blood specimens, IL-6 and TAS activity decreased significantly in the DM group compared with the control group (*p* = 0.016 and *p* = 0047, respectively). However, the TNF-a, MDA, TOS, and OSI results were not statistically significant (*p* > 0.05). The TAS and OSI values were also statistically significant in the DM + Q treatment group compared with the DM group (*p* = 0.016 and 0.009, respectively). In the DM + P + Q group, only IL-6 differed significantly from the group with DM-related damage (*p* = 0.018) (Table 2). Examination of tissue specimens in terms of oxidant and antioxidant parameters and pro-inflammatory cytokines revealed a significant increase in MDA levels in the DM group compared with the control group (*p* = 0.028), while no significant differences were determined in TAS, TOS, OSI, SOD, CAT, or GSH activities (*p* > 0.05). MDA levels and SOD activity decreased significantly in the DM + P group compared with the DM group (*p* = 0.016 and *p* = 0.025, respectively). The MDA and CAT levels also decreased significantly in the DM + Q group compared with the DM group (*p* = 0.016 and 0.028, respectively). Finally, the MDA, SOD, and TOS values decreased significantly in the DM + P+ Q treatment group compared with the DM group (*p* = 0.045, *p* = 0.009, and *p* = 0.011, respectively). The SOD activity in the combined treatment group (DM + P + Q) and was close to that of the control group (Table 3).

## 4. Discussion

DPN is one of the most widespread chronic complications of diabetes, and there is a strong probability of DPN developing in approximately half of patients during the course of the disease [5]. Glycemic variability has been linked to the pathogenesis of complications during the prediabetic process and is reported to be an independent risk factor for the onset of metabolic disorders underlying DPN [25,26]. Some of the first findings reported in short- and long-term diabetes studies include an increase in glucose levels and loss of body weight [27,28,29]. Although several therapeutic options are available, no ideal therapeutic protocol for diabetes-related neuropathy has been described. New and effective therapeutic strategies are therefore urgently required, and although the popularity of the use of natural products is growing rapidly due to their multi-mechanistic effects [30,31], the focus has recently been placed on glycemic control for preventing the development of DPN and improving the management thereof [32].

Bee products are regarded as a rich source of biologically active compounds. Propolis is a resinous secretion collected by honeybees from the buds or sap streams of specific trees or other botanical sources and is employed as a traditional medicine in many countries [33,34]. In vitro studies of propolis have stressed that it may exhibit an anti-hyperglycemic property due to its inhibitory effect on a-amylase and a-glucosidase activities. Nda et al. (2021) reported that the four-week application of propolis reduced hyperglycemia by lowering blood sugar in STZ-induced DM [30]. Another study reported that six-week 100 mg/kg and 200 mg/kg propolis supplementation exhibited a lowering effect on blood glucose levels [33]. Quercetin is a natural polyhydroxyflavonoid found in the flowers, leaves, and fruits of edible plants such as onions, apples, lettuce, and cabbage. A previous study showed that it reduced blood glucose concentrations in rats and mice in an experimentally induced model of diabetes and preserved β-cell numbers in the pancreas and insulin sensitivity [35]. Another important characteristic is that it lowers aldose reductase, an enzyme that converts glucose to sorbitol via the polyol pathway [36]. Ojo et al. (2021) reported that a four-week quercetin administration significantly reduced blood sugar in an STZ-induced model of diabetes [37]. Elbe et al. (2015) investigated the effects of quercetin on STZ-induced DM complications and observed that it reduced blood sugar levels and produced a marked improvement in body weight at the end of 30 days [38]. Consistent with previous research, in the present study, a decrease in body weight was observed in rats with STZ-induced DM at the end of the four-week experimental period, together with a pronounced increase in fasting blood sugar levels. While no improvement in body weight occurred in the DM + P group, blood sugar levels decreased. In the DM + Q and DM + P + Q treatment groups, blood sugar levels decreased markedly, and body weight also increased. The particularly notable finding was that body weight in the group with combined propolis and quercetin treatment was close to that of the control group and was more compatible with the fasting blood sugar regulation process. In addition, the occurrence of diarrhea in the DM + P group may have slightly affected the weight loss/gain measurements and may be the reason why body weight was close to the control group in the group that received combined propolis and quercetin treatment. Thus, quercetin may be an ameliorator of the deleterious effects of dose-based propolis. We think that the synergetic effects of propolis and quercetin in DM are more positive in terms of stabilizing blood sugar levels and body weight. Since the loss of body weight in diabetic rats is associated with muscle wastage due to excessive catabolism of tissue proteins as a result of hyperglycemia, the synergetic effect of administered antioxidants may be sufficient to maintain body weight through the control of blood glucose, thus preventing protein catabolism [38].

Although the pathogenesis of DPN is complex due to the multifactorial causes underlying the condition, chronic hyperglycemia is the principal cause of metabolic diseases and is also an important factor in intensive oxidative stress in diabetes. Increased oxidative stress following hyperglycemia is principally responsible for auto-oxidative glycosylation and advanced glycation end products. This affects the increasing activity of the polyol pathway, subsequently leading to sorbitol accumulation and increased intracellular osmolarity and oxidative stress [27,31]. Oxidative stress is thought to function as a biochemical trigger for sciatic nerve dysfunction, as a result of which, increased cellular ROS causes membrane lipid peroxidation, protein nitration, and degradation of DNA, associated with the course of DPN [38]. MDA is a characteristic compound of the oxidative stress process. Enzymes such as SOD, CAT, and glutathione peroxidase (GSH-Px) in cells are responsible for antioxidant defense. TAS, TOS, and OSI are the factors reflecting the redox balance between oxidation and antioxidation. TAS is a marker of the activity of all antioxidants, while TOS is a marker of ROS. The OSI represents the ratio of TOS to TOS and indicates oxidative stress levels [17]. Wang et al. (2020), Addepalli et al. (2018), and Khan et al. (2022) reported that MDA levels increased significantly while SOD, CAT, and GSH activity decreased in STZ-induced diabetic rats [27,28,39]. A different study examined TOS and TAS values in sciatic nerve tissue for the evaluation of neuro-oxidative stress and observed a significant increase in the DM group. A loss of equilibrium between ROS production and antioxidant defense systems, particularly with an elevated oxidative stress index, has been reported to lead to neuronal damage [40]. Sayın et al. (2014) reported significantly lower TAS values in neuropathic patients with type 2 DM compared with healthy controls and higher MDA levels in diabetic neuropathic patients than in healthy controls [41]. The analysis of oxidant/antioxidant and proinflammatory cytokine parameters in the present study revealed a marked increase in MDA levels and a significant decrease in IL-6 and TAS values in the DM group. However, the results for TNF-a, TOS, and OSI were not statistically significant. The study findings appear to partly support those examined in the literature. This raises the possibility that the damage mechanism may not have occurred directly through oxidative stress.

Propolis is rich in various compounds, the variety of which depends on the honeybee species, the plant source involved, the climatic conditions, and the harvesting season [10,42]. At least 300 components are present in the propolis complex. Phenolic compounds and esters, flavonoids (such as flavonols, flavones, flavanones, dihydroflavonols, and chalcones), terpenes, aromatic acids, aromatic aldehydes and alcohols, sesquiterpenes, and caffeic phenylester (CAPE) are among propolis organic compounds [11]. Propolis exhibits a number of different biological activities, including antibacterial, anti-fungal, antiviral, antioxidant, anti-inflammatory, cytotoxic, immunomodulatory, neuro-protective, antihepatotoxic, and antitumor effects. Studies have shown that the flavonoids and related compounds found in propolis are the compounds with the highest free radical scavenging activity [43]. It also protects against various cancers including head, lung, liver, brain, kidney, and prostate cancer [44]. Preclinical animal studies have confirmed the effectiveness of propolis on glucose, lipid metabolism, insulin, and antioxidant activity [8]. Propolis has also been shown to induce the activation of antioxidant enzymes including CAT and SOD against free radicals [17]. Brazilian green propolis has been reported to significantly reduce TNF levels but not to interfere with other interleukins. Kanbur et al. (2009) reported that propolis reduces oxidative stress by suppressing MDA levels in plasma and various organs while exhibiting a partial effect on antioxidant enzymes such as SOD, CAT, and GSH-Px [11]. Scientific studies have also confirmed the reported biological advantages of quercetin, such as anti-inflammatory, antioxidant, antidiabetic, anti-atherosclerotic, and analgesic properties [45,46,47]. One of the most potent antioxidant flavonoids, quercetin, exhibits its effect either directly through ROS inhibition or through the induction of the activities of antioxidant enzymes in the endogenous defense system [37,47]. Due to these characteristics, quercetin has also been used in the treatment of coronary heart disease, neurodegenerative diseases, various types of cancer, and various inflammatory conditions [47]. Previous studies investigating the effects of quercetin on antioxidant enzyme levels have reported that SOD improves oxidative stress in the brain and that quercetin administered for three weeks significantly reduced lipid peroxide levels in rats. Quercetin has been described as reducing MDA and neural nitrite levels, preventing falls in neural SOD and GSH levels following diabetes, and lowering TNFα and IL-1β levels in the sciatic nerve [48]. Another study reported that the neuroprotective mechanism of quercetin in cerebral ischemia caused a reduction in metalloproteinase-9 and lowered free radical production [18]. The difference in TAS and OSI between the DM + Q treatment group and the DM group in the present study was statistically significant. MDA levels and SOD activity decreased significantly in the DM + P group compared with the DM group. MDA and CAT levels decreased significantly in the DM + Q group compared with the DM group. Significant decreases were also observed in MDA, IL-6, and TOS values in the DM + P + Q treatment group compared with the DM group. SOD activity in the combined treatment group was close to that of the control group. We believe that propolis and quercetin combination is important for SOD activity. In terms of the evaluation of treatment groups among themselves, we think that the combined effect of propolis and quercetin may be more effective in the oxidant/antioxidant balance and the control of inflammatory cytokines.

Peripheral system pathological changes such as axonal atrophy and degeneration and demyelination findings have been observed in an STZ-induced model of DM [49]. Hyperglycemia has been strongly implicated in the DPN mechanism. Axonal atrophy, segmental demyelination, and fiber loss have been observed at ultrastructural examinations in an STZ-induced DM model fiber, and research has emphasized that axonal atrophy may be associated with osmotic shrinkage or delayed axonal development [26]. Degeneration of the axon and myelin sheath, separation in the concentric lamellar structure of the myelin sheath, axonal swelling, vacuoles of various sizes between the myelin sheath, and occasional invagination of the myelin sheath into the axon were observed during ultrastructural examinations and on semi-thin sciatic nerve sections from rats with STZ-induced diabetes in the present study. Morphometric evaluations revealed marked decreases in myelin sheath fiber numbers and myelin sheath thickness. Our histopathological findings are consistent with data from previous STZ-based DN models. In terms of duration, DPN, described as a chronic complication following DM, resulted in morphological damage to the sciatic nerve within a 28-day period and impaired the normal axonal maturation process. Morphometric and ultrastructural examinations of tissue specimens from the DM + P, DM + Q, and DM + P + Q treatment groups showed that degenerated areas in sciatic nerve fibers, were replaced by regeneration, that active axonal maturation and remyelination were processed, and that increases occurred in the myelin fiber thickness and myelinated nerve fiber numbers. When all the treatment groups were compared among themselves, a significant improvement was observed in DM damage findings in the combined propolis and quercetin group (DM + P + Q). Studies investigating the neuroprotective effects of propolis have noted that it exhibits ameliorating effects on sensory–motor functions in cerebral artery occlusion, that it improves motor functions and the sciatic functional index in sciatic nerve injuries, and that it increases myelinated nerve fibers [39,50]. Nosratiyan et al. (2021) and Saho et al. (2022) reported that propolis can be used as an alternative supplement in regenerative medicine since it results in axon myelination and contributes to progenitor cell differentiation [51,52]. Quercetin is a natural flavonoid with antidiabetic properties that is capable of crossing the blood–brain barrier. It is known to exhibit protective effects against neuronal damage deriving from hyperglycemia [53]. Chis et al. (2017) emphasized that moderate-intensity aerobic exercises with 30 mg/kg quercetin improved sciatic nerve function and reduced histopathological damage in STZ-diabetic rats [54]. Another study involving rat diabetic models showed that 20 and 40 mg/kg quercetin administration over eight weeks could significantly improve nerve conduction velocity in motor and sensory nerves [48]. Xe et al. (2020) administered 50 mg/kg quercetin for 12 weeks to rats with STZ-induced DPN and reported that ultrastructural examination of the sciatic nerve revealed significant improvement in the myelin sheath surrounding the axon at the morphological and morphometric levels [55]. When the effects of separate administrations of propolis and quercetin were evaluated in the present study, the morphological results supported research in the previous literature (at different doses and periods).

Molecular studies have revealed abnormal expression of inflammatory cytokines in the microvasculature around the peripheral nerves in patients with diabetic neuropathy and in various animal models [56]. Nerve damage initiates an inflammatory response and induces the expression of pro-inflammatory cytokines including TNF-α, IL-1β, and IL-6 [50]. The upregulation of TNF-α promotes an inflammatory response leading to nerve dysfunction, which results in inflammatory damage and nerve cell demyelination [57]. In addition, DM-mediated inflammation leads to cell/tissue damage by sending apoptotic signals. The clinicopathological symptoms of diabetic neuropathy are nerve fiber demyelination and myelin damage induced by the apoptosis of Schwann cells. These play an important role in high glucose-derived damage and in accelerating axonal demyelination, and permanent diabetes and glucose elevation result in sciatic nerve myelin damage and Schwann cell apoptosis. Quercetin may also play a protective role in nerve cells in neuronal structures, the anti-inflammatory response, antioxidant stress, and the inhibition of apoptosis [57]. Twenty-eight-day quercetin administration in crush-type injuries has been reported to play an active role in the regeneration process of the sciatic nerve [18]. The diffuse effects of propolis include amelioration of inflammation and apoptosis in different experimental models [58]. Inflammation and apoptosis were evaluated using TNF-α, IL-1β immunoreactivities, and the TUNEL technique in the present study. The results revealed significant increases in AI, TNF-α, and IL-1β immunopositivity scores in the DM group, while immunoreactivity and apoptosis in Schwan cells decreased markedly in all the treatment groups. Based on our own findings and the previous literature, we think that the separate and combined application of propolis and quercetin play an ameliorating role through their anti-inflammatory and antiapoptotic effects.

## 5. Conclusions

In conclusion, the separate applications of propolis and quercetin on STZ-induced diabetic peripheral neuropathy damage appear to play an active role in the regulation of metabolic processes and signaling pathways. These can also be useful natural products for ameliorating the neurological complications of diabetes due to their anti-inflammatory, antioxidant, and neuroprotective properties. In addition, our scan of the literature revealed no previous studies investigating the combined effects of the combined use of propolis and quercetin at doses of 100 mg/kg. There is a strong likelihood that when the two adjuvants are applied in combination, they may make a positive contribution to nerve function by yielding the most effective, optimal morphological healing. In terms of the damage mechanism and particularly, blood sugar regulation, they may represent a key alternative supplement in regenerative medicine for ensuring glucose regulation and preventing chronic complications since the primary cause of pathogenicity is hyperglycemia.

## Figures and Tables

**Table 1:** Blood glucose levels and body weight of the experimental group.

Group	Blood Glucose Level (mg/dL)					Body Weight (g)
	Initial	First Week	Second Week	Third Week	Forth Week	
Control	89.33 ± 9.00	92 ± 9.20	94.83 ± 8.20	88.16 ± 3.37	87.83 ± 9.38	372.66 ± 22.28
P	84.66 ± 5.31	106.33 ± 12.27	99.33 ± 7.33	95.83 ± 8.10	90.33 ± 8.26	384.33 ± 32.61
Q	93.83 ± 11.25	102.83 ± 14.77	100.33 ± 8.14	93.16 ± 7.65	92.16 ± 6.24	389.83 ± 31.57
P + Q	97.33 ± 11.27	95.66 ± 18.81	94.33 ± 6.12	101.66 ± 7.86	98.16 ± 5.49	359.83 ± 41.74
DM	421.66 ± 78.17 ^a^	501.83 ± 137.38 ^a^	424.33 ± 48.19 ^a^	415.16 ± 55.14 ^a^	409.66 ± 16.13 ^a^	235.33 ± 41.73 ^a^
DM + P	439.5 ± 49.24 ^a^	421.66 ± 52.24 ^a^	390.16 ± 35.04 ^a^	357.66 ± 31.70 ^a.b^	297.33 ± 24.84 ^a.b^	301.33 ± 46.96 ^a^
DM + Q	448.16 ± 82.49 ^a^	401.66 ± 7.94 ^a^	369.83 ± 17.98 ^a^	350 ± 28.40 ^a.b^	271 ± 33.39 ^a.b^	334.5 ± 27.32 ^b^
DM + P+ Q	431.5 ± 89.12 ^a^	367.66 ± 50.14 ^a.b^	388.5 ± 34.09 ^a^	353 ± 46.67 ^a.b^	255.66 ± 27.69 ^a.b.c^	357.33 ± 36.60 ^b^

Data are expressed as mean ± standard deviation. P; propolis, Q; quercetin, DM; diabetes mellitus. ^a^; *p* < 0.05 compared with the control group. ^b^; *p* < 0.05 compared with the DM group. ^c^; *p* < 0.05 compared with the DM + P group.

**Table 2:** Biochemical findings for serum samples in all groups.

Group	MDA (µmol/mL)	TOS (μmol H_2_O_2_ Eqv./L)	TAS (μmol Trolox Eqv./L)	OSI (AU)	TNF-α (pg/mL)	IL-6 (pg/mL)
Control	3.56 ± 0.49	19.57 ± 5.90	1.15 ± 0.12	1.717 ± 0.55	6.55 ± 1.46	5.19 ± 2.92
P	2.22 ± 0.59	19.03 ± 11.01	1.18 ± 0.15	1.65 ± 1.00	4.93 ± 1.70	28.93 ± 3.21
Q	4.87 ± 1.43	17.63 ± 11.04	1.31 ± 0.18	1.35 ± 0.80	5.61 ± 1.42	31.06 ± 2.54
P + Q	3.43 ± 0.95	20.08 ± 9.82	1.36 ± 0.61	1.61 ± 0.84	4.83 ± 2.32	29.65 ± 3.81
DM	6.74 ± 1.89 ^a^	24.66 ± 7.71	0.82 ± 0.22	3.31 ± 1.84	5.55 ± 3.01	28.82 ± 11.63 ^a^
DM + P	8.57 ± 1.60	18.27 ± 6.81	0.98 ± 0.30	1.98 ± 0.98	4.48 ± 1.5	40.84 ± 6.43
DM + Q	7.62 ± 1.51	18.80 ± 1.27	1.21 ± 0.12 ^b^	1.56 ± 0.12 ^b^	5.74 ± 0.84	34.96 ± 3.86
DM + P + Q	6.78 ± 0.89	18.44 ± 6.79	0.75 ± 0.21	2.66 ± 1.33	4.23 ± 1.73	31.90 ± 3.86 ^b^

Data are expressed as mean ± standard deviation. P; Propolis, Q; Quercetin, DM; Diabetes mellitus. ^a^; *p* < 0.05 compared to the control group. ^b^; *p* < 0.05 compared to the DM group.

**Table 3:** Biochemical findings for sciatic nerve tissue samples in all groups.

Group	MDA Tissue (nmol/mg)	TOS Tissue (μmol H_2_O_2_ Eqv./mg Protein)	TAS Tissue (μmol Trolox Eqv./mg Protein)	OSI Tissue (AU)	SOD Units/mg Protein	CAT Units/mg Protein	GSH µg/mg Protein
Control	3.87 ± 1.50	72.94 ± 20.31	5.40 ± 2.08	1.83 ± 1.70	3.15 ± 2.29	37.92 ± 19.93	265.87 ± 115.29
P	2.04 ± 1.40	70.84 ± 9.55	5.73 ± 1.21	1.25 ± 0.17	3.27 ± 1.78	30.29 ± 17.29	171.89 ± 62.27
Q	3.36 ± 1.68	66.56 ± 15.24	5.59 ± 1.24	1.22 ± 0.34	3.00 ± 0.42	19.31 ± 4.94	189.55 ± 125.62
P + Q	3.26 ± 0.63	60.24 ± 9.78	4.44 ± 2.84	1.93 ± 1.15	3.44 ± 0.83	27.65 ± 13.99	194.58 ± 94.25
DM	7.02 ± 1.92 ^a^	83.74 ± 13.37	2.87 ± 0.72	3.13 ± 1.16	5.04 ± 0.19	17.94 ± 6.05	230. 86 ± 86.95
DM + P	4.00 ± 0.99 ^b^	60.80 ± 30.44	3.07 ± 1.37	2.60 ± 2.11	3.64 ± 0.34 ^b^	28.10 ± 15.96	266.82 ± 177.88
DM + Q	3.09 ± 1.54 ^b^	71.57 ± 8.62	3.56 ± 3.5	3.12 ± 1.60	6.90 ± 1.69	38.88 ± 26.11 ^b^	227.22 ± 198.57
DM + P + Q	4.13 ± 1.62 ^b^	49.55 ± 15.15 ^b^	3. 03 ± 1.17	1.82 ± 0.77	3.40 ± 1.17 ^b^	19.01 ± 17.02	325.81 ± 139.63

Data are expressed as mean ± standard deviation. P; propolis, Q; quercetin, DM; diabetes mellitus. ^a^; *p* < 0.05 compared with the control group. ^b^; *p* < 0.05 compared with the DM group.

## Data Availability

Data is contained within the article.
